# Supplementary material for: Asynchronous magnetic resonance elastography: Shear wave speed reconstruction using noise correlation of incoherent waves
Source: Magn Reson Med. 2022 Oct 27;89(3):990–1001. doi: 10.1002/mrm.29502 (PMC9792433; doi:10.1002/mrm.29502)
Supplement: Supplementary file 1 — DATA S1 MATLAB code used to generate the numerical simulations of Figure 1 [file MRM-89-990-s002.zip › k-Wave/helpfiles/makeLine.html]

makeLine :: Functions (k-Wave)


# makeLine

Create a binary map of a straight line within a 2D grid.

## Syntax

```
line = makeLine(Nx, Ny, startpoint, endpoint)
line = makeLine(Nx, Ny, startpoint, angle, length)
```

## Description

`makeLine` creates a binary map of a line within a two-dimensional grid (the line is denoted by 1's in the matrix with 0's elsewhere). The line can be defined either by its endpoints or by a starting point, an angle (direction), and a length.

## Inputs

|  |  |
| --- | --- |
| `Nx, Ny` | size of the 2D grid [grid points] |
| `startpoint` | two-element vector identifying the start point for the line [grid points] |
| `endpoint` | two-element vector identifying the end point for the line [grid points] |
| `angle` | the direction of the line [radians] |
| `length` | the length of the line [grid points] |

## Outputs

|  |  |
| --- | --- |
| `line` | 2D binary map of a line |

## See Also

`makeArc`, `makeCircle`, `makeDisc`
